# Evaluation of a CBT-Based Program for Mental Health in the General Population During the COVID-19 Pandemic: A Stepped-Care Approach Using a Chatbot and Digitized Group Intervention

**DOI:** 10.1155/2024/8950388

**Published:** 2024-11-26

**Authors:** Till Langhammer, Kevin Hilbert, Robert Wasenmueller, Berit Praxl, Andrea Ertle, Julia Asbrand, Ulrike Lueken

**Affiliations:** ^1^Department of Psychology, Humboldt-Universität zu Berlin, Berlin, Germany; ^2^Department of Psychology, HMU Health and Medical University Erfurt, Erfurt, Germany; ^3^Mental Tech LLC (GmbH), Berlin, Germany; ^4^Department of Psychology, Friedrich Schiller University Jena, Jena, Germany; ^5^German Center for Mental Health (DZPG), Partner Site Berlin/Potsdam, Berlin, Germany

**Keywords:** anxiety, chatbot, COVID-19, depression, digital mental health intervention, group intervention, stepped-care

## Abstract

**Background:** The COVID-19 pandemic exposed a substantial portion of society to multiple stressors, while access to mental health care was limited. To address this, we introduced a digital stepped-care program rooted in cognitive–behavioral therapy (CBT) principles, aiming to alleviate mental health distress among the general public seeking help.

**Methods:** The program comprises a 4-week digital application using “Aury” the chatbot, followed by an optional 6-week online group session for those still symptomatic. A 4-week waiting period separated these steps. Participants entered based on self-identified mental health concerns. Interventions addressed prevalent pandemic mental health issues: sleep disturbances, anxiety, depression, worry/rumination, interpersonal issues, and resource mobilization. Outcomes focused on depressive, anxiety, and somatic symptoms, assessed by the Patient Health Questionnaire (PHQ).

**Results:** Of the 1261 initial participants, postchatbot results (*N* = 142) indicated small to medium effects (*d* = 0.412 to *d* = 0.523). Those finishing the entire program (*N* = 41) saw substantial symptom decline with medium to large effects (*d* = 0.757 to *d* = 0.818). No shifts were seen in the waiting phase. At follow-up 6 months after baseline, both groups—those who only used the chatbot (*N* = 60; *d* = 0.284 to *d* = 0.416) and those who completed the entire program (*N* = 27; *d* = 0.854 to *d* = 0.926)—showed sustained symptom reduction. Comparing groups that received no intervention, used the chatbot only, and completed the entire program, we observed a dose–response effect.

**Conclusions:** This resource-efficient and adaptable digital approach effectively reduced pandemic-induced mental health issues, indicating its potential in crisis periods with limited health resources. Randomized controlled trials are recommended for further validation.

**Trial Registration:** Clinical Trial Registry identifier: DRKS00023220.

## 1. Introduction

The COVID-19 pandemic, both through its direct effects and indirectly via containment measures, impacted societies, leading to significant mental stress among large segments of the population [1]. This has been reflected in a notable increase in mental health problems, as evidenced by various meta-analyses encompassing data from different countries worldwide [2–5]. These studies have consistently shown a significant rise in anxiety, depression symptoms, and sleep problems as immediate responses to the pandemic. A recent meta-analysis estimated that the global impact of the pandemic led to an additional 53 million cases of major depressive disorder and 76.2 million cases of anxiety disorders [6].

Compounding the issue, access to mental health care has been limited due to the implementation of containment measures. Consequently, there has been an urgent need for easily accessible, resource-efficient, and scalable interventions to alleviate the mental health burden and prevent the development of mental disorders in those seeking care [2, 7, 8].

Indicated prevention involves targeted interventions aimed at preventing the development or worsening of a specific health condition in individuals at high risk, such as those experiencing persistent but subclinical symptoms of stress. Numerous studies investigating indicated prevention for depression and anxiety disorders have demonstrated the benefits of both online and in-person interventions [9, 10]. These interventions have proven successful in targeting anxious and depressive thoughts [9].

Digital approaches to mental health are especially viable and appropriate for reaching large numbers of people at low thresholds, particularly during periods of social distancing and containment measures [11, 12]. They also minimize the risk of potential COVID-19 infections for both participants and clinicians. Automatic digital interventions, in particular, are easily accessible and highly scalable, making them well-suited as the initial step in a stepped-care program during a pandemic. Subsequent interventions at higher intensities can then be tailored as a second step based on individual responses and symptom profiles.

Stepped care, as a model of healthcare delivery, is particularly well-suited for indicated prevention. It involves providing progressively intensifying care based on the individual's needs and preferences. In practice, it begins with easily accessible, low-threshold interventions for a broad population, followed by more intensive care for individuals who do not initially respond or continue to report symptoms. The impact of the pandemic has varied among individuals, influenced by risk (e.g., young age, female gender, prepandemic mental illness) and protective factors (e.g., emotional regulation competencies, social support, availability of accurate information; [2, 13, 14]). As a result, individuals at risk may require different levels of psychological support, making stepped-care programs a resource-efficient solution, particularly in the context of limited healthcare resources.

To address the lack of psychological support programs in response to the mental health burden of the pandemic, we developed a digital mental health stepped-care program based on cognitive–behavioral therapy (CBT) as indicated prevention. The program was designed to be easily accessible, tailored to the pandemic situation, and offered different levels of support through the stepped-care approach. It consisted of two program components:

1. Automated chatbot “Aury”: Participants engaged with an automated chatbot that provided support and guidance. This component targeted common mental health issues experienced during the pandemic, including sleep problems, anxiety symptoms, worry/rumination, depressive symptoms, interpersonal conflicts, and resource activation.
2. Digitized group intervention: Participants who reported persistent mental health problems were admitted to this step. Led by trained clinical psychologists, this digitized group intervention provided additional support and addressed the same mental health concerns as step 1, but in a manner tailored to the specific needs of the participants.

The two program steps were separated by a 4-week waiting period.

Our objective was to assess the immediate effects and the effects at a 6-month follow-up (6M-FU) of the program on key mental health problems, namely depressive, anxiety, and somatic symptoms.

We hypothesized that the automated chatbot, delivering psychoeducational content and practical exercises, would lead to a significant reduction in depression, anxiety, and somatic symptoms. Additionally, we hypothesized that the digitally delivered group intervention would provide a more in-depth and personalized treatment experience, resulting in additional reductions in depression, anxiety, and somatic symptoms. In contrast, we did not expect that symptoms would change during the 4-week waiting period as this period did not involve any active intervention. For the 6M-FU, we expected the effects of the program to be temporarily stable, indicating that the benefits gained during the intervention would persist over time. Furthermore, we hypothesized a positive relationship between treatment dose and symptom improvement. We exploratively analyzed subjective ratings of both interventions to gain insights into participants' experiences and perceptions of the program's effectiveness.

## 2. Materials and Methods

### 2.1. Study Design

This study was conducted as part of the intervention “Stress-free after Corona: a psychological support program of the Humboldt-Universität zu Berlin.” Detailed information about the study methods can be found in Langhammer et al. [15], which provides a comprehensive overview of the study design and procedures.

**Figure 1:**
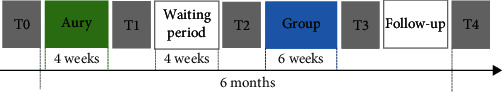
Design. Assessment time points in gray; intervention step 1 (chatbot) in green; intervention step 2 (group) in blue; waiting time in white; T0—baseline and before Aury; T1—post Aury; T2—after 4-week waiting period and before online group; T3—post online group; and T4—follow-up 6 months after baseline.

Ethical considerations regarding low-threshold access and the provision of immediate support led to the choice of a nonrandomized controlled design, building upon a within-subject 4-week waiting period which was implemented between the two program steps. As shown in Figure 1, study assessments were conducted at five-time points.

The design also included an ongoing data collection for FU assessments at 12 and 24 months after T0 (not reported here). The study was conducted in accordance with the Declaration of Helsinki and approved by the Ethics Committee of the Institute of Psychology, Humboldt-Universität zu Berlin (No. 2020-35). The entire program was free of charge for all participants.

### 2.2. Participants

To be eligible for study enrollment, participants needed to meet the following inclusion criteria: proficiency in the German language (since the program was delivered in German) and having the ability to provide informed consent. For step 2 of the program, participants were enrolled if they demonstrated clinically significant symptom burden, indicated by a score above the cutoff (>10 points) on at least one of the subscales (PHQ-9, GAD-7, PHQ-15; [16–19]) of the Patient Health Questionnaire (PHQ), or if they self-reported a subjective need for the group intervention. This report includes solely adult participants aged 18 years and older, as there were low participation rates among adolescents.

While participation in the automated chatbot was open to everyone, there were exclusion criteria for the digital group intervention. Participants with evidence of severe mental disorders that would impede their ability to engage in a group setting (e.g., signs of acute psychosis or acute suicidality) were excluded. Experienced clinical psychologists conducted screening video or phone calls with all participants interested in participating in the group intervention to assess their suitability for the intervention.

Participants were recruited through various channels, including local and national press, flyers, social media platforms, university mailing lists, student unions, psychotherapy outpatient clinics, and medical practices in Berlin. The enrollment period spanned from September 1, 2020 to April 15, 2021.

**Figure 2:**
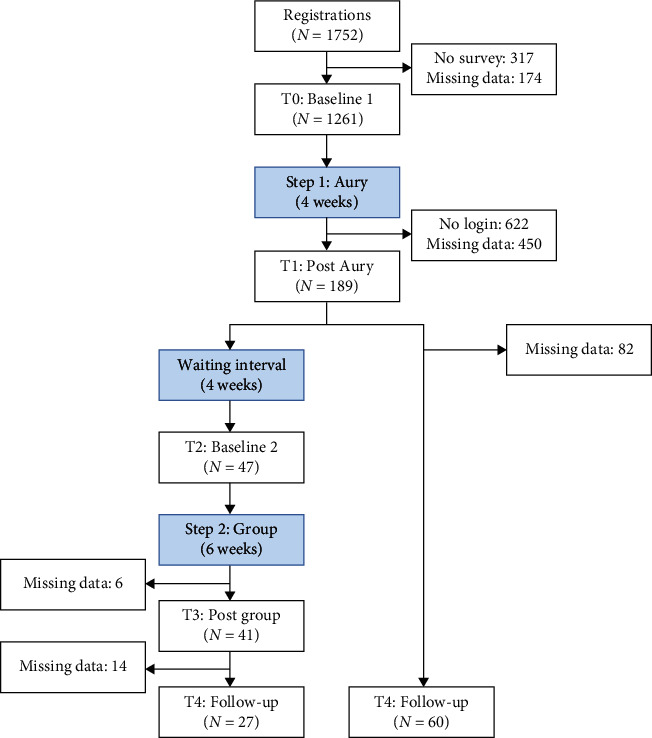
Flowchart.

Figure 2 illustrates the study's progression. After registering for the program, most participants completed the baseline assessment (T0). Those who registered for the chatbot received an email with a link to the first postassessment (T1) after 4 weeks in which they were supposed to use the chabot. Participants who continued to meet the cutoff in at least one scale, or who were interested regardless of this, qualified for the second step of the intervention. After a 4-week waiting period following T1, they received another email with a link to complete the second baseline assessment (T2). Upon completing the group intervention, they received a link to the second post-assessment (T3). Finally, all participants were sent a FU assessment link 6 months after the baseline assessment (T4).

### 2.3. Digital CBT-Based Stepped-Care Program

Aury is an algorithm-based automatic chatbot that delivers content based on CBT principles. It offers 24 modules covering topics such as sleep problems, anxiety, depression, worry/rumination, interpersonal conflicts, and resource activation (5–10 min for each module). Participants could personalize their experience by choosing the sequence and number of modules to complete. In addition, six longer modules could be individualized to a specific problem that participants wanted to work on, following the principles of solution-focused brief therapy [20]. Aury was available free of charge for 4 weeks and can still be accessed online (https://aury.so/ for an updated version).

The online group intervention provided greater treatment intensity and individualization compared to the chatbot. It followed a manualized approach and focused on the same topics as the chatbot. Sessions were conducted online once per week for 100 min (standard length of a double therapy session in the German health care system) over 6 weeks, with a maximum of 10 participants per group. The sessions included CBT techniques, psychoeducation, behavior modification, and activation exercises. Progressive muscle relaxation according to Jacobson [21] was initiated and resource-focused tasks were completed each session. Participants in need of additional help were offered individual sessions at the outpatient clinic for anxiety disorders at Humboldt-Universität zu Berlin.

#### 2.3.1. Documentation of Intervention Participation and Adherence

Concerning the utilization of the chatbot, user behavior was automatically recorded. Active participation (completer sample) was determined by showing at least one login to the chatbot and completion of the postintervention assessment (T1). Intent-to-treat (ITT) was defined as users who had logged into the chatbot at least once.

For the group intervention, we collected data on participation and ensured compliance with the online sessions. To ensure adherence, all clinicians who conducted the groups underwent training using a standardized manual developed by our group (available upon request). They were also offered weekly supervision by the project supervisors. Completion for the entire program was defined as attending the intervention at least once and completing the postintervention assessment (T3). ITT was defined as participants expressing their intention to start the group by completing the preassessment (T2).

### 2.4. Assessments

The entire assessment battery is given in Langhammer et al. [15] and Hilbert et al. [13] and included sociodemographic variables, quality of life, psychopathology, family stress, coping, and pandemic-related stress. As primary outcomes, we used a German translation of the PHQ-D (version C) as a dimensional (severity of symptoms) and categorical (cutoffs for a clinically relevant syndrome) measure of depression, anxiety, and somatic symptoms. The version C of the PHQ-D is an established instrument with three subscales (nine items for depressive symptoms—PHQ-9, 15 items for somatic symptoms—PHQ-15, and seven items for anxiety/panic symptoms—GAD-7) and good psychometric properties [17, 18, 22, 23].

### 2.5. Statistical Analyses

We utilized a completer and an ITT approach (last observation carried forward [LOCF]). Furthermore, we investigated the association between changes observed from pre- to postintervention and the level of program utilization as the dose–response effect. This enabled us to explore how participants' engagement with the program influenced their experienced outcomes.

To evaluate the immediate effects of the chatbot Aury only (T0–T1, hypothesis 1) and the entire program (T0–T3, hypothesis 2) on depressive, anxiety, and somatic symptoms, we conducted paired samples *t*-tests. Additionally, we performed *t*-tests to determine if any effects observed beyond the Chatbot intervention (T1–T3) were solely due to the online group intervention. This analysis involved comparing symptom levels before and after the waiting period (T1–T2, hypothesis 3), as well as comparing symptom levels before and after the online group intervention (T2–T3).

For follow-up effects, we performed *t*-tests to assess the sustainability of effects following postassessments for both the chatbot Aury only (T1–T4, hypothesis 4) and the entire program (T3–T4).

For both immediate and FU effects, response rates were analyzed using McNemar's *χ*^2^-test.

To examine the dose–response effects of program utilization, we conducted one-way analyses of covariances (ANCOVAs) with planned linear contrasts. These contrasts were employed to compare pre–post differences among groups, differentiated by the intensity of their program engagement. A covariate was incorporated to account for initial symptom severity. We categorized participants into three groups: those who attended the postassessment (T0–T1) without engaging in any intervention, those who solely used Aury (T0–T1), and those who completed the entire program (T0–T3).

Additionally, subjective assessments of the interventions' effects are presented descriptively, and where appropriate, Cohen's *d* effect sizes were calculated. All analyses were performed with the statistical software Statistical Package for Social Sciences (SPSS) 26.0 (IBMCorp), *p*  < 0.05 was considered statistically significant.

## 3. Results

As given in Figure 2, of the 1752 individuals who were eligible and enrolled in the study, 1261 completed the baseline assessment. For characterization and comparison of the baseline sample with the general population, see Hilbert et al. [13]. To investigate the immediate effects of the respective interventions, 189 complete records from Aury-users (142 Aury-only users, that did not participate in the second intervention) and 41 complete records from participants who completed the entire program were available. To investigate the effects after the 6M-FU, 60 records of Aury-only users (who did not participate in the group intervention) and 27 records of those who completed the entire program were available. ITT findings are given in the Supporting Information. For descriptive statistics see Table 1 (and Table S1 for ITT sample).

### 3.1. Immediate and FU Effects

Statistics are given in Table 2. Immediate effects of Aury (T0–T1): The completer analysis revealed a significant decrease in depressive, anxiety, and somatic symptoms after 4 weeks of Aury usage in both samples: all Aury users (*N* = 189) and those exclusively using Aury (*N* = 142). These effects were characterized by small effect sizes.

Immediate effects of the entire program (T3–T0): The completer analysis revealed a significant decrease in depressive, anxiety, and somatic symptoms among participants who completed the entire program (*n* = 41). These effects were characterized by medium effect sizes. Furthermore, when examining the differential effects of Aury and the group intervention within those who used Aury and attended the group, several findings emerged. After adjusting for multiple comparisons, no significant changes in depressive and anxiety symptoms were observed during the period of Aury use or the waiting period. However, small effect sizes were observed for the group intervention. However, significant medium effect sizes were found for Aury in relation to somatic symptoms, while no change was observed during the waiting period and group intervention.

**Figure 3:**
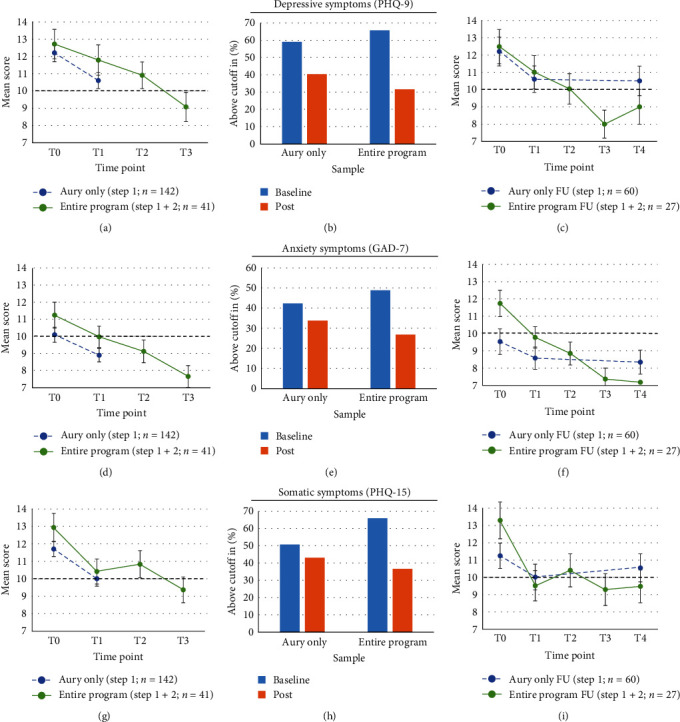
(a, d, and g) Pre- and post-intervention changes at four time points before (T0) and after the first intervention Aury (T1) and both interventions entire program (T3) with a 4-week waiting period (T1–T2; means and standard errors). (b, e, and h) Percentage of participants above the clinical cutoff before and after participation. (c, f, and i) Follow-up subsample with all five time points including changes from postassessments (T1 and T3) and follow-up 6 months after baseline (T4; means and standard errors). Black dotted lines—clinical cutoff, indicating a mental disorder.

The response rate for immediate effects in terms of the changes in proportions of individuals exceeding the clinical cutoff was statistically significant across all scales and samples. The only exception was for anxiety symptoms following Aury, both for the “Aury only” group and the “Aury completer” group. For more detailed statistical information, please refer to Table 2 (and Table S2 for ITT sample) and Figure 3.

In the ITT analyses, significance did not change, whereas effect sizes for the chatbot were smaller (see Supporting Information for more details).

Figure 3 and Table 3 illustrate that the trajectories of a subsample from the FU assessment align with those of the pre–post sample. Nevertheless, some observed changes did not achieve statistical significance, possibly because of the constraints of the sample sizes.

FU effects of Aury (T4–T1): The effects on all three outcomes remained stable for those who exclusively used Aury, during the FU period of up to 6 months after the baseline assessment. Here, the effect on anxiety symptoms did not reach significance (after correcting for multiple comparisons).

FU effects of the entire program (T4–T3): The effects on all three outcomes persisted consistently from the postassessment to the FU after completing the entire program. While the overall FU effects were significant for all three scales, only the changes in depressive symptoms, observed before and after participation in the online group (the second step), achieved significance in this limited subsample.

Here, the changes in proportions of individuals exceeding the clinical cutoff were only statistically significant across all scales after the entire program. For more detailed statistical information, please refer to Table 2 and Figure 3.

In the ITT analyses, depressive and anxiety symptoms decreased during follow-up, but not somatic symptoms (see Supporting Information for more details).

### 3.2. Dose–Response Effects

**Figure 4:**
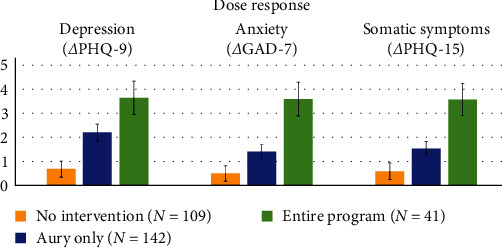
Pre–post differences in all three subscales (means and standard error). GAD-7, General Anxiety Disorder Scale-7; PHQ-9, Patient Health Questionnaire-9; PHQ-15, Patient Health Questionnaire-15.

There was a linear trend in differences in depressive symptoms (*F* (2, 289) = 27.152, *p* < 0.001), anxiety symptoms (*F* (2, 289) = 22.145, *p* < 0.001), and somatic symptoms (*F* (2, 289) = 19.235, *p* < 0.001; see Figure 4) after controlling for baseline symptom severity. This suggests that as the intensity of program use increased, there was a proportional reduction in these symptoms.

### 3.3. Subjective Evaluation

**Figure 5:**
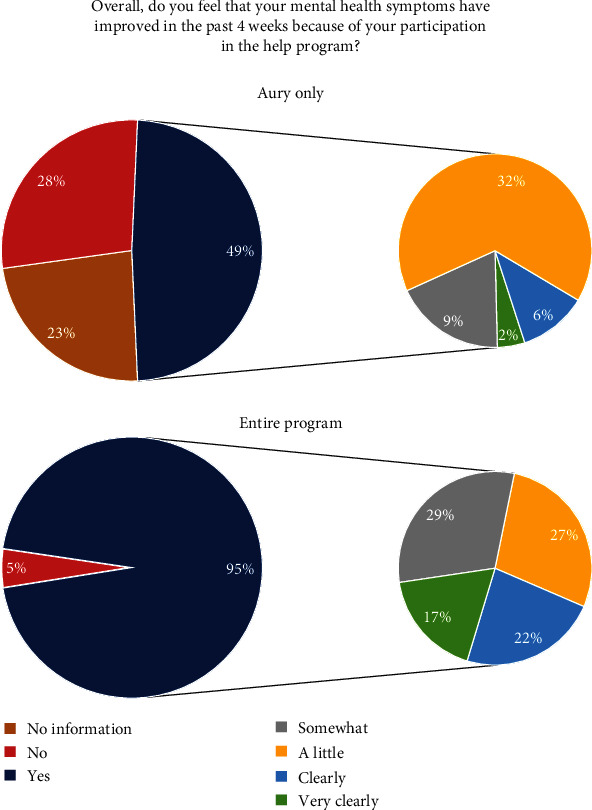
Subjective attribution of improvement in psychological distress.

Descriptive analyses of the evaluation results reveal that 49% of participants found our program beneficial after using Aury, and this number significantly increased to 95% after the completion of the entire program, as illustrated in Figure 5.

## 4. Discussion

The COVID-19 pandemic has significantly impacted psychological well-being and mental health, thereby highlighting the urgent need for scalable, low-threshold digital interventions. We here introduced a novel digital mental health stepped-care program based on CBT principles to address mental health issues under conditions of a pandemic. It offered a stepped-care approach, tailored to individual needs, to prevent mental health problems from evolving into chronic conditions. The main findings were as follows: (I) The automated chatbot Aury effectively reduced depressive, anxiety, and somatic symptoms, with effects ranging from small to medium. (II) Participants who completed the entire program demonstrated medium to large effect sizes. (III) These effects remained consistent throughout the 6-month FU period. (IV) A dose–response relationship was evident, with increased treatment intensity leading to greater symptom reduction.

As hypothesized, both the chatbot and the online group resulted in significant symptom reductions. Interestingly, a sizable number of participants found significant benefit from the chatbot alone, bringing their symptom scores down to subthreshold levels. Our findings also suggest that the chatbot intervention had a comparably strong effect on somatic symptoms for those completing the entire program, despite these symptoms not being our primary target. We propose this might be an indirect effect, where reducing depressive and anxiety symptoms subsequently alleviates somatic symptoms. Initial or considerable improvement in psychological health could potentially lead to improved physical well-being. A comprehensive meta-analysis by He et al. [24] assessed the efficacy of conversational agent interventions (CAIs; chatbots utilizing algorithms and machine learning, akin to Aury) on various mental health challenges [24]. The study showed that CAIs had a small to medium impact in alleviating depressive, anxiety, and somatic symptoms. Another meta-analysis by Lim et al. [25] similarly reported medium effect sizes for chatbot interventions targeting depression [25]. The effect sizes observed at FU by He et al. [24] were notably diminished (to either no or small effects), averaging around half the magnitude of the short-term effects. The outcomes varied based on the target sample (whether clinical and symptomatic nonclinical samples were examined combined or separately) and the control condition (as various control conditions were explored). It becomes evident that our chatbot, despite addressing a broad range of internalizing issues rather than a single specific issue, generates comparably effective results, and in some areas, even surpasses the efficacy of existing interventions within a relatively short time frame. Furthermore, within our FU period, effects were stable in opposition to those reported in He et al. [24].

Participants who engaged in the entire program—using both the chatbot and participating in the online group sessions—showed substantial symptom reduction above the effect of the first intervention and across all measures with small to medium effect sizes for the online intervention and medium to large effect sizes for the entire program. This corroborates our hypothesis on the added value of the group intervention. The rapidly expanding field of online interventions for mental disorders has spurred numerous systematic reviews and meta-analyses, as researchers navigate this multifaceted landscape of diverse studies. Multiple meta-analyses on digital interventions (independent of the way of communication via chatbot or other ways) for depressive and anxiety symptoms, targeting diverse populations (both preventive and therapeutic), have been conducted. Most consistently, they report small to medium effect sizes for both prevention and treatment of depressive [26–30] and anxiety symptoms [28, 31, 32]. Interestingly, two meta-analyses report large effect sizes for both depression and anxiety when examining internet-based full-blown CBT therapy for clinically diagnosed patients [33, 34]. This contrasts with internet-based interventions that aim to reduce symptoms in heterogenous target groups using CBT and other psychotherapy-oriented methods. Among those that distinguish between guided and unguided interventions, either none of both or the guided approaches appear more effective, showcasing medium effect sizes compared to the smaller effects of unguided ones. Twomey et al. [35] conducted a meta-analysis aimed at evaluating the effect size of a specific long-established digital intervention, Deprexis, which has demonstrated efficacy in the treatment of depression [35]. Their findings revealed a medium effect size, consistent across different target populations (patients vs. the general community) and irrespective of guidance (whether with or without professional assistance). Digital interventions seem to be helpful and effect sizes mostly depend on target group and specificity of the intervention. During the COVID-19 pandemic, in a meta-analysis by Ye, Li, and Zhu [36], it was found that psychosocial interventions effectively alleviated depressive and anxiety symptoms, producing medium and large effect sizes respectively [36]. This analysis included studies that targeted diverse groups such as the general public, health workers, students, and more. However, when outliers were removed in a subanalysis, the average effect size reduced to a small level for depressive and anxiety symptoms, comparable to those in meta-analyses before the pandemic. It is worth noting that the effects at FU were considerably weaker than the short-term results. Subgroup analyses indicated that studies incorporating CBT or those with a passive control group showed medium to large effect sizes. In summary, our approach of integrating both unguided and guided digital interventions in a stepped-care format appears successful. Each intervention individually achieved effect sizes in line with those reported in existing literature, whereas studies in the literature differ concerning the way effect sizes were measured (pre–post, between group, and other ways). Furthermore, the FU effects of our program hold promise. Nonetheless, it is worth noting that traditional in-person CBT continues to exhibit larger effect sizes compared to its digital counterparts [27].

Our initial hypotheses did not distinguish between participants who continued with the program after using the chatbot and those who did not. Given our results, we can conclude that those who continued to be above the cutoff after the first intervention, or who independently wanted to join the group, not only showed an absolute value above 10 in at least one measure but also benefitted fundamentally less from the chatbot in terms of change. This further underscores the merit of our stepped-care approach.

In the ITT analysis for chatbot Aury, even though more than twice as many participants did not complete the postassessment (thus showing no change), the results still held significance. This underscores the robustness of the findings. However, the effect sizes were smaller. Among those who completed the entire program, the sample differences were minimal, and the outcomes remained consistent, further underscoring the strength of the results. Contrary to this, slight variations were noted in the FU ITT analyses. Symptoms of depression and anxiety decreased post–chatbot interaction, but somatic symptoms remained unchanged. For participants who completed the entire program, symptoms remained stable, consistent with the completer analyses. Overall, the ITT analysis showed only slight deviations, primarily in effect sizes.

Furthermore, the linear trend in symptom reduction—from no intervention, to chatbot use only, to participation in the full program—indicates a dose–response relationship, supporting a causal effect of the intervention on subsequent symptom reduction again, even without a control group. When surveyed, 49% of participants indicated they benefited from the program after using the chatbot, while 95% saw benefits after completing the entire program. Considering the initial motivation behind the program—to support individuals during periods of limited health care availability—these findings are particularly significant.

### 4.1. Strengths and Limitations

The program was designed to offer immediate psychological support to all interested participants, eliminating the possibility of introducing a waiting list or placebo control condition. Moreover, a stepped-care approach was adopted to best match the immediate needs of the situation. While our program shows encouraging outcomes, it is crucial to interpret these results with caution. The absence of a control condition and randomization, coupled with varying sample sizes between the steps, complicates the interpretation of the findings. Still, the absence of effect during a waiting period and a dose–response effect within our program points to a distinct intervention impact beyond mere spontaneous remission. To further substantiate these preliminary results, a study employing a randomized controlled design is essential.

It is worth noting that the sample was self-selective, rather than specifically targeted patients, and does not reflect the broader population accurately. At baseline, more than 74.5% of the program's participants surpassed thresholds for clinically significant conditions such as depression, anxiety, and somatization. As reported early in a study by Hilbert et al. [13], the majority of participants were female and well-educated, mirroring the often-observed bias in psychotherapy studies [13]. This aligns with the trend of women seeking mental health care more frequently. This overrepresentation might be due to a disproportionate number of participants coming from our academic environment, even though this was not our primary recruitment channel. Since participants were not aware of the chatbot's specific content beforehand, the initial appeal was likely not influenced by program details. We approached recruitment without focusing on any specific gender. This means our efforts were not biased toward any gender. To improve generalizability, future studies should explore strategies to better engage underrepresented groups, particularly males, to design more inclusive mental health interventions.

Furthermore, it is important to note the high nonstarter rate between registration and the initial step. One potential reason could be that some of the informational material offered by the chatbot and in the group was accessible on the registration webpage. Adherence rates vary considerably across different digital interventions and can even fluctuate within the same type of intervention. Fleming et al. [37] conducted a study exploring the uptake and engagement with freely accessible digital self-help interventions for depression, low mood, or anxiety, revealing a broad range of adherence rates from 21% to 88% for minimum usage, defined as logging in at least once post-registration [37]. In our specific self-help intervention, the adherence rate, measured in terms of the percentage of users with minimal usage, was observed to be 36.47%, which is consistent within the range reported in the reviewed interventions. Drop-out of therapy is a common concern among clinicians, with ~19.7% of clients discontinuing treatment early, a rate varying across specific demographics and settings [38]. However, the drop-out rate for the second step of our program (online group) was notably low at 10.67%, lower than the average drop-out rate typically observed in conventional psychotherapy.

Despite these limitations, the program's main strengths lie in its resource efficiency and scalability, making it particularly valuable during pandemics and community crises. Additionally, its manualized structure simplifies the learning process for clinical psychologists, facilitating its wider dissemination in clinical practice.

## 5. Conclusion

Our stepped-care program utilized a digital, resource-efficient design with variable intensity and diverse content crosscutting traditional diagnostic boundaries, hence being suitable for a large number of participants. The program has demonstrated both feasibility and efficacy, reaching a broad audience with minimal resources via a fully online platform that demonstrates a significant dose–response effect. As we move into an era of increasing digitization and limited access to conventional psychotherapy resources, such digital interventions are essential to address a wide range of at-risk persons or bridge long waiting times for psychotherapeutic treatment [39, 40].

Integrating digital content into primary care, the stepped-care approach addresses the common issue of low adherence to standalone digital health interventions. By offering personalized face-to-face interventions when necessary as a second step, it could enhance motivation and compliance. The program has the potential for addressing mental health needs during crises and periods of limited mental healthcare resources, like waiting times for outpatient psychotherapy. Therefore, it presents itself as a scalable, resource-efficient solution.

Before adopting this program more broadly, it is crucial to perform confirmatory testing through a randomized controlled design. As current times are characterized by multiple global crises, adaptable and scalable digital interventions will be increasingly important to bridge mental health care gaps and therefore fostering societal resilience.

## Figures and Tables

**Table 1:** Demographic and clinical characteristics of the baseline and completer samples.

	All participants (*n* = 1261)		Aury only (*n* = 142)		Entire program (*n* = 41)		Aury only vs. entire program	
	Mean	SD	Mean	SD	Mean	SD	*t*/*χ*^2^	*p*
Demographic characteristics								
Female gender (*n* [%])	1002	(79.5)	118	(83.1)	35	(85.4)	0.119	0.730
Age	41.97	(13.46)	41.32	(12.19)	39.39	(13.20)	0.878	0.381
User behavior “Aury”								
Number of logins	—	—	3.33	(4.79)	4.71	(5.33)	−1.580	0.116
Completed modules	—	—	1.15	(1.86)	2.32	(2.65)	−3.204	0.002
Clinical characteristics								
Depressive symptoms	11.32	(5.71)	12.25	(6.12)	12.71	(5.44)	−0.429	0.669
Above cutoff (*n* [%])	599	(47.50)	84	(59.15)	27	(65.85)	0.598	0.439
Anxiety symptoms	9.74	(5.00)	9.87	(5.26)	11.24	(4.88)	−1.488	0.139
Above cutoff (*n* [%])	731	(58.00)	60	(42.25)	20	(48.78)	0.551	0.458
Somatic symptoms	11.26	(5.38)	11.24	(5.17)	12.93	(5.16)	−1.843	0.067
Above cutoff (*n* [%])	617	(48.93)	72	(50.70)	27	(65.85)	2.940	0.087

Abbreviations: GAD-7, anxiety symptoms; PHQ-9, depressive symptoms; PHQ-15, somatic symptoms; SD, standard deviation.

**Table 2:** Statistical results for the treatment pre–post effects, separated for Aury-only (step 1), and the entire program.

		Paired differences					*t*	*df*	*p*	FDR corrected *p*^a^	Cohen's *d*	Above cutoff baseline in (%)	Above cutoff post in (%)	*χ* ^2^	*p*
		Mean	SD	SE	95% confidence interval of the difference										
					Lower	Upper									
Aury (*N* = 189)															
PHQ-9	Pre–post	−1.841	4.235	0.308	−1.234	−2.449	−5.978	188	<0.001^*∗∗*^	<0.001^*∗∗*^	0.435	67.72	52.91	18.225	<0.001^*∗∗*^
GAD-7	Pre–post	−1.254	3.568	0.260	−0.742	−1.766	−4.831	188	<0.001^*∗∗*^	<0.001^*∗∗*^	0.351	50.79	42.86	1.362	0.121
PHQ-15	Pre–post	−1.746	3.667	0.267	−1.220	−2.272	−6.546	188	<0.001^*∗∗*^	<0.001^*∗∗*^	0.476	61.38	50.79	7.848	0.005^*∗*^

Aury only (*N* = 142)															
PHQ-9	Pre–post	−2.190	4.189	0.352	−2.885	−1.495	−6.231	141	<0.001^*∗∗*^	<0.001^*∗∗*^	0.523	59.15	40.48	12.971	<0.001^*∗∗*^
GAD-7	Pre–post	−1.401	3.400	0.285	−1.966	−0.837	−4.911	141	<0.001^*∗∗*^	<0.001^*∗∗*^	0.412	42.23	33.80	1.531	0.108
PHQ-15	Pre–post	−1.528	3.552	0.298	−2.118	−0.939	−5.126	141	<0.001^*∗∗*^	0.010^*∗∗*^	0.430	50.70	43.00	3.781	0.026^*∗*^

Entire program (*N* = 41)															
PHQ-9	Pre–post	−3.634	4.646	0.726	−5.101	−2.168	−5.008	40	<0.001^*∗∗*^	<0.001^*∗∗*^	0.782	65.85	31.71	10.563	0.001^*∗*^
	Pre–post: Aury	−0.927	4.401	0.687	−2.316	0.462	−1.348	40	0.185	0.202	0.211	—	—	—	—
	Waiting period	−0.878	3.716	0.580	−2.051	0.295	−1.513	40	0.138	0.166	0.236	—	—	—	—
	Pre–post: online group	−1.829	3.626	0.566	−2.974	−0.685	−3.231	40	0.002^*∗*^	0.006^*∗*^	0.505	—	—	—	—

GAD-7	Pre–post	−3.585	4.738	0.740	−5.081	−2.090	−4.845	40	<0.001^*∗∗*^	<0.001^*∗∗*^	0.757	48.78	26.8	3.765	0.025*⁣*^*∗*^
	Pre–post: Aury	−1.268	3.988	0.623	−2.527	−0.010	−2.037	40	0.048^*∗*^	0.073	0.318	—	—	—	—
	Waiting period	−0.854	3.321	0.519	−1.902	0.195	−1.646	40	0.108	0.143	0.257	—	—	—	—
	Pre–post: online group	−1.463	3.887	0.607	−2.690	−0.237	−2.411	40	0.021^*∗*^	0.041^*∗*^	0.377	—	—	—	—

PHQ-15	Pre–post	−3.561	4.353	0.680	−4.935	−2.187	−5.238	40	<0.001^*∗∗*^	<0.001^*∗∗*^	0.818	65.85	36.60	8.471	0.001^*∗*^
	Pre–post: Aury	−2.512	4.013	0.627	−3.779	−1.245	−4.008	40	0.000^*∗∗*^	0.001^*∗∗*^	0.626	—	—	—	—
	Waiting period	0.415	3.840	0.600	−0.796	1.627	0.691	40	0.493	0.493	0.108	—	—	—	—
	Pre–post: online group	−1.463	4.160	0.650	−2.776	−0.150	−2.253	40	0.030^*∗*^	0.051	0.352	—	—	—	—

Abbreviations: FDR, false discovery rate; GAD-7, General Anxiety Disorder Scale-7 (anxiety symptoms); PHQ-9, Patient Health Questionnaire-9 (depressive symptoms); PHQ-15, Patient Health Questionnaire-15 (somatic symptoms); SD, standard deviation; SE, standard error.

^a^Calculated per sample.

*⁣* ^*∗*^Means statistical significance *p* < 0.001.

*⁣* ^*∗∗*^Means statistical significance *p* < 0.05.

**Table 3:** Statistical results for the treatment follow-up effects, separated for Aury-only (step 1), and the entire program.

		Paired differences					*t*	*df*	*p*	FDR corrected *p*^a^	Cohen's *d*	Above cutoff baseline in (%)	Above cutoff post in (%)	*χ* ^2^	*p*
		Mean	SD	SE	95% confidence interval of the difference										
					Lower	Upper									
Aury only (*N* = 60)															
PHQ-9	Pre-FU6M	−1.600	3.850	0.497	−2.594	−0.606	−3.219	59	0.002^*∗*^	0.012^*∗*^	0.416	63.33	56.67	0.643	0.212
	Post-FU6M	−0.100	4.646	0.600	−1.300	1.100	−0.167	59	0.868	0.868	0.022	—	—	—	—

GAD-7	Pre-FU6M	−0.950	3.347	0.432	−1.815	−0.085	−2.199	59	0.032^*∗*^	0.064	0.284	43.33	41.67	0.000	1.000
	Post-FU6M	−0.233	3.280	0.423	−1.081	0.614	−0.551	59	0.584	0.701	0.071	—	—	—	—

PHQ-15	Pre-FU6M	−1.233	3.605	0.465	−2.165	−0.302	−2.650	59	0.010^*∗*^	0.030^*∗*^	0.342	51.67	55.00	0.071	0.395
	Post-FU6M	0.533	3.615	0.467	−0.400	1.467	1.143	59	0.258	0.387	0.148	—	—	—	—

Entire program (*N* = 27)															
PHQ-9	Pre-FU6M: entire program	−4.481	4.839	0.931	−6.396	−2.567	−4.813	26	<0.001^*∗∗*^	<0.001^*∗∗*^	0.926	74.07	37.04	8.100	<0.001^*∗∗*^
	Pre–post: Aury	−1.481	5.064	0.975	−3.485	0.522	−1.520	26	0.141	0.235	0.293	—	—	—	—
	Waiting period	−0.963	3.838	0.739	−2.481	0.555	−1.304	26	0.204	0.255	0.251	—	—	—	—
	Pre–post: online group	−2.037	3.848	0.741	−3.559	−0.515	−2.751	26	0.011^*∗*^	0.033^*∗*^	0.529	—	—	—	—
	Post-FU6M	1.000	3.258	0.627	−0.289	2.289	1.595	26	0.123	0.231	0.307	—	—	—	—

GAD-7	Pre-FU6M: entire program	−4.370	4.789	0.922	−6.265	−2.476	−4.742	26	<0.001^*∗∗*^	<0.001^*∗∗*^	0.913	59.26	25.93	5.818	0.006^*∗*^
	Pre–post: Aury	−1.963	4.301	0.828	−3.664	−0.262	−2.372	26	0.025^*∗*^	0.063	0.456	—	—	—	—
	Waiting period	−0.926	3.529	0.679	−2.322	0.470	−1.363	26	0.184	0.251	0.262	—	—	—	—
	Pre–post: online group	−1.481	4.423	0.851	−3.231	0.268	−1.740	26	0.094	0.201	0.335	—	—	—	—
	Post-FU6M	−0.185	3.270	0.629	−1.479	1.109	−0.294	26	0.771	0.826	0.057	—	—	—	—

PHQ-15	Pre-FU6M: entire program	−4.000	4.682	0.901	−5.852	−2.148	−4.439	26	<0.001^*∗∗*^	<0.001^*∗∗*^	0.854	81.48	44.44	8.100	<0.001^*∗∗*^
	Pre–post: Aury	−3.778	3.906	0.752	−5.323	−2.233	−5.026	26	<0.001^*∗∗*^	<0.001^*∗∗*^	0.967	—	—	—	—
	Waiting period	0.889	3.816	0.734	−0.621	2.399	1.210	26	0.237	0.273	0.233	—	—	—	—
	Pre–post: online group	−1.111	4.173	0.803	−2.762	0.539	−1.384	26	0.178	0.267	0.266	—	—	—	—
	Post-FU6M	0.185	3.530	0.679	−1.211	1.582	0.273	26	0.787	0.787	0.052	—	—	—	—

Abbreviations: FDR, false discovery rate; GAD-7, General Anxiety Disorder Scale-7 (anxiety symptoms); PHQ-9, Patient Health Questionnaire-9 (depressive symptoms); PHQ-15, Patient Health Questionnaire-15 (somatic symptoms); SD, standard deviation; SE, standard error.

^a^Calculated per sample.

*⁣* ^*∗*^Means statistical significance *p* < 0.001.

*⁣* ^*∗∗*^Means statistical significance *p* < 0.05.

## Data Availability

Data cannot be shared publicly as this is not included in the informed consent by participants and the mental health data is particularly sensitive. However, deidentified participant data with annotations will be made available to other researchers upon reasonable request (to the study administration at corona-stressfrei.psychologie@hu-berlin.de). Additionally, a registered study protocol is available at the German Clinical Trial Register (https://www.drks.de), and a methods paper describing the overall research project is also available [15].

## Supporting Information

Supporting Information Table S1. Demographic and clinical characteristics of the baseline and intent-to-treat samples. Table S2. Pre–post and in-depth analyses of the program.
