# Supplementary material for: Evaluation of a CBT-Based Program for Mental Health in the General Population During the COVID-19 Pandemic: A Stepped-Care Approach Using a Chatbot and Digitized Group Intervention
Source: Depress Anxiety. 2024 Nov 26;2024:8950388. doi: 10.1155/2024/8950388 (PMC11919055; doi:10.1155/2024/8950388)
Supplement: Supporting Information — Table S1. Demographic and clinical characteristics of the baseline and intent-to-treat samples. Table S2. Pre–post and in-depth analyses of the program. [file 8950388.f1.docx]

**Supplemental material**

**Results**

To investigate the immediate effects of the respective interventions in an intent-to-treat approach (ITT), 637 complete records from Aury-users (587 Aury-only users, that did not participate in the second intervention) and 46 complete records from participants who completed the entire program (attended both interventions) were available. To investigate the effects after the 6M-FU, 105 records of Aury-only users (who did not participate in the group intervention) and 31 records of those who completed the entire program were available. For descriptive statistics see Table 1.

Table S1: Demographic and clinical characteristics of the baseline and intent-to-treat samples.

|  | No intervention (n = 1261) | | Aury only (n = 587) | | Entire program (n = 46) | |
| --- | --- | --- | --- | --- | --- | --- |
| *Demographic characteristics* |  |  |  |  |  |  |
| Female gender [n(%)] | 1002 | (79.5) | 486 | (82.8) | 38 | (82.6) |
| Age | 41.97 | (13.46) | 40.53 | (12.94) | 38.85 | (12.81) |
| *User behaviour “Aury”* |  |  |  |  |  |  |
| Number of Logins^1^ |  |  | 2.19 | (3.30) | 4.52 | (5.23) |
| Completed Modules^1^ |  |  | .58 | (1.37) | 2.17 | (2.56) |
| *Clinical characteristics* |  |  |  |  |  |  |
| Depressive symptoms (PHQ-9) | 11.32 | (5.71) | 11.54 | (5.55) | 12.80 | (5.19) |
| Above cut-off [n(%)] | 599 | (47.50) | 318 | (54.17) | 32 | (69.57) |
| Anxiety (GAD-7) | 9.74 | (5.00) | 10.01 | (4.84) | 10.83 | (4.92) |
| Above cut-off [n(%)] | 731 | (58.00) | 255 | (43.44) | 21 | (45.65) |
| Somatic symptoms (PHQ15) | 11.26 | (5.38) | 11.38 | (5.24) | 12.78 | (5.05) |
| Above cut-off [n(%)] | 617 | (48.93) | 312 | (53.15) | 30 | (65.22) |

Note: Depressive symptoms – PHQ-9, anxiety symptoms – GAD-7, somatic symptoms – PHQ-15.

### 3.1 Immediate and follow-up effects

Immediate effects of Aury (T1-T0): The impact of using Aury was evaluated by comparing baseline measurements (T0) with post-intervention measurements (T1). The ITT analysis revealed a significant decrease in depressive, anxiety, and somatic symptoms after four weeks of Aury usage in both samples: all Aury users (n=637) and those exclusively using Aury (n=587). These effects were characterized by very small effect sizes. The last observation carried forward approach lead to similar values at baseline and post assessment for the majority of participants.

Immediate effects of the entire program (T3-T0): The impact of the entire program was evaluated by comparing baseline measurements (T0) with post-intervention measurements (T3). The ITT analysis revealed a significant decrease in depressive, anxiety, and somatic symptoms among participants who completed the entire program (n=46). These effects were characterized by medium effect sizes. Furthermore, when examining the differential effects of Aury and the group intervention within those who used Aury and attended the group, several findings emerged. Specifically, after adjusting for multiple comparisons, no significant changes in depressive and anxiety symptoms were observed during the period of Aury use or the waiting period. However, small effect sizes were observed for the group intervention. Moreover, significant medium effect sizes were found for Aury in relation to somatic symptoms, while no change was observed during the waiting period and group intervention. For more detailed statistical information, please refer to Table S2.

Table S2: Pre-post and in-depth analyses of the program.

|  | | Paired Differences | | | | | t | df | *p* | FDR corrected *p^1^* | Cohen's d |
| --- | --- | --- | --- | --- | --- | --- | --- | --- | --- | --- | --- |
|  |  | Mean | Std. Deviation | Std. Error Mean | 95% Confidence Interval of the Difference | |  |  |  |  |  |
|  |  |  |  |  | Lower | Upper |  |  |  |  |  |
| **Aury completer**  **(N = 637)** |  |  |  |  |  |  |  |  |  |  |  |
| Depressive symptoms | Pre-post | -0,546 | 2,451 | 0,097 | -0,737 | -0,356 | -5,625 | 636 | .000** | .000** | .099 |
| Anxiety symptoms | Pre-post | -0,372 | 2,023 | 0,080 | -0,529 | -0,215 | -4,642 | 636 | .000** | .000** | .078 |
| Somatic symptoms | Pre-post | -0,518 | 2,147 | 0,085 | -0,685 | -0,351 | -6,089 | 636 | .000** | .000** | .099 |
|  |  |  |  |  |  |  |  |  |  |  |  |
| **Aury only completer (N =587)** |  |  |  |  |  |  |  |  |  |  |  |
| Depressive symptoms | Pre-post | -0,530 | 2,259 | 0,093 | -0,713 | -0,347 | -5,682 | 586 | .000** | .000** | .097 |
| Anxiety symptoms | Pre-post | -0,339 | 1,773 | 0,073 | -0,483 | -0,195 | -4,633 | 586 | .000** | .000** | .071 |
| Somatic symptoms | Pre-post | -0,370 | 1,862 | 0,077 | -0,521 | -0,219 | -4,811 | 586 | .000** | .000** | .070 |
|  |  |  |  |  |  |  |  |  |  |  |  |
| **Entire Program**  **(n = 46)** |  |  |  |  |  |  |  |  |  |  |  |
| Depressive symptoms | Pre-post: Entire program | -3,435 | 4,792 | 0,707 | -4,858 | -2,012 | -4,862 | 45 | .000** | .000** | .632 |
|  |  |  |  |  |  |  |  |  |  |  |  |
|  | Pre-post: Aury | -0,739 | 4,276 | 0,630 | -2,009 | 0,531 | -1,172 | 45 | .247 | .270 | .137 |
|  | Waiting period | -1,065 | 3,708 | 0,547 | -2,166 | 0,036 | -1,948 | 45 | .058 | .086 | .197 |
|  | Pre-post: Online Group | -1,630 | 3,466 | 0,511 | -2,660 | -0,601 | -3,190 | 45 | .003* | .006* | .299 |
|  |  |  |  |  |  |  |  |  |  |  |  |
| Anxiety symptoms | Pre-post: Entire program | -3,087 | 5,037 | 0,743 | -4,583 | -1,591 | -4,157 | 45 | .000** | .001* | .673 |
|  |  |  |  |  |  |  |  |  |  |  |  |
|  | Pre-post: Aury | -0,890 | 4,064 | 0,599 | -2,076 | 0,337 | -1,451 | 45 | .154 | .184 | .195 |
|  | Waiting period | -0,913 | 3,265 | 0,481 | -1,883 | 0,056 | -1,897 | 45 | .064 | .086 | .219 |
|  | Pre-post: Online Group | -1,304 | 3,693 | 0,545 | -2,401 | -0,208 | -2,395 | 45 | .021* | .042* | .303 |
|  |  |  |  |  |  |  |  |  |  |  |  |
| Somatic symptoms | Pre-post: Entire program | -3,391 | 4,409 | 0,650 | -4,701 | -2,082 | -5,216 | 45 | .000* | .000* | .687 |
|  |  |  |  |  |  |  |  |  |  |  |  |
|  | Pre-post: Aury | -2,304 | 3,943 | 0,581 | -3,475 | -1,133 | -3,963 | 45 | .000* | .001* | .478 |
|  | Waiting period | 0,217 | 3,898 | 0,575 | -0,940 | 1,375 | 0,378 | 45 | .707 | .707 | .045 |
|  | Pre-post: Online Group | -1,304 | 3,949 | 0,582 | -2,477 | -0,132 | -2,240 | 45 | .030* | .052 | .264 |

^1^calculated per subsamp

Follow-up effects of Aury (T4-T1): Depressive and anxiety symptoms decreased from post assessment to 6M-FU for those who exclusively used Aury (depressive symptoms t(104) = -2.466, p = .015, *d* = .207; anxiety symptoms t(104) = -2.611, p = .010, *d* = .230), whereas somatic symptoms remained stable (somatic symptoms t(59) = -.715, p = .476).

Follow-up effects of the entire program (T4-T3): The effects on all three symptom scales remained stable after attending the entire program (depressive symptoms t(30) = 1.751, p = .090; anxiety symptoms t(30) = -.288, p = .776; somatic symptoms t(30) = .710, p = .483) during the follow-up period of up to 6 months after the baseline assessment.
